# Golden Rules

**Published:** 1894-02

**Authors:** 


GOLDEN RULES.—Never tap a suspected renal tumor through the abdominal parietes, *i. e.*, through the peritoneum. Never think lightly of any ulcer of the tongue or lips of a patient beyond middle life. Never permit a healthy wet-nurse to suckle a syphilitic child, or child of syphilitic parents. Never neglect to warn your patient about his eyes in treating a first "attack" of gonorrhea. Never forget many gleets are due to slight contractions of the canal and may be cured by a steel bougie.—*Med. Rec.*

